# Online housing search dataset: Information flows of real estate platform users

**DOI:** 10.1016/j.dib.2021.107327

**Published:** 2021-08-27

**Authors:** Joep Steegmans, Jonathan de Bruin

**Affiliations:** a Department of Economics, Leiden University, Steenschuur 25, Leiden 2311 ES, the Netherlands; b Utrecht School of Economics, Utrecht University, P.O. Box 80125, Utrecht 3508 TC, the Netherlands; c Research and Data Management Services, Utrecht University, Heidelberglaan 8, Utrecht 3584 CS, the Netherlands

**Keywords:** User-generated data, Housing market, Real estate platform, Online search, Gravity modeling

## Abstract

This data article describes user-generated data of Funda.nl, the largest online housing market website of the Netherlands. The data contain the inflow and outflow of hits (mouse clicks, opening of webpages, etc.) at the municipality level. The municipality of the user defines the origin and the municipality of the property that is viewed defines the destination. The data capture real behavior of the platform users. The flow data are based on 1.1 billion hits that are made by the users of the website in the first six months of 2018. The underlying data are collected by Google Analytics, the web analytics tool of Google. Funda utilizes the data for platform stability, security, product development, etc. The proprietary data of Funda are used to generate the information flows between municipalities. In the full sample we have 148,216 information flows between municipalities in the Netherlands, among which 313 zero flows. The data include subsamples for different types of platform users as user search intentions range from serious to fully recreational. The data enable researchers to analyze housing search behavior from a novel perspective. The data are, for instance, relevant for housing market researchers, digital economists, and economic geographers.

## Specifications Table

**Table:** 

Subject	Economics, Econometrics and Finance
Specific subject areas	Real Estate Economics, Big Data Analytics, Economics, Geography
Type of data	Table
How data were acquired	The data originate from Google’s web analytics tool, Google Analytics, which is used by Funda to register website traffic. Tables are created in the Google Cloud with BigQuery [1], which allows querying with standard SQL.
Data format	Mixed (raw, preprocessed, derived) Analyzed
Parameters for data collection	The information flow data are based on proprietary data of Funda. The data collection complies with the General Data Protection Regulation (GDPR), which protects data and privacy of individuals in Europe. The information flow data cannot be linked to individual platform users. Funda has agreed upon publication of these flow data. Nevertheless, Funda has had no influence, directly or indirectly, in the construction of the datasets.
Description of data collection	The underlying data originate from Google Analytics, which is used by Funda to register website traffic. BigQuery is used to query the data and create the datasets described in this data article.
Data source location	Owner underlying web analytics: Funda Real Estate B.V., Amsterdam, the Netherlands. The flow data capture information flows within the Netherlands. The hits in the flows are made by platform users from the Netherlands on objects (i.e., houses and apartments) in the Netherlands.
Data accessibility	Repository name: Mendeley Data Dataset: J. Steegmans, J. de Bruin, Online housing search dataset: Information flows of real estate platform users, Mendeley Data, V1, 2021. DOI: https://doi.org/10.17632/rtn8t47t6j.1[2] URL: https://doi.org/10.17632/rtn8t47t6j.1
Related research article	J. Steegmans, J. de Bruin, Online housing search: A gravity model approach, PLOS ONE 16(3): e0247712 (2021). DOI: 10.1371/journal.pone.0247712[3].

## Value of the Data

• The data provide novel information on (online) housing search. The information flows are based on housing search activity of housing platform users. The data differentiate between serious searchers and recreational searchers, so that they can be compared and analyzed.
• Researchers can use the datasets to gain insights in housing search processes, both online and offline. The data are particularly suited for studies that try to explain bilateral flows between locations, for instance through so-called gravity models. Beyond housing market applications the data are valuable for researchers interested in information flows in general.
• From a cross-sectional perspective, these datasets seem amongst the most extensive in existence for gravity model applications (for a thorough literature overview, see [4]). Missing values and zero flows are relatively rare as the flows are based on big data, i.e., over a billion user hits.
• The data demonstrate the research potential of novel data sources, such as user-generated platform data. What is more, the data demonstrate that web analytics of online platforms can be transformed into meaningful datasets for academic use.
• It is likely that housing search flow data can be used to predict future residential mobility. Therefore, these data seem valuable in, for instance, land-use and spatial planning.

## 1 Data Description^1^

### 1.1 Information flows

The information flow datasets, or hit flow datasets, described in this data article are based on user-generated data of Funda.nl, the largest online housing market website of the Netherlands. The Dutch Association of Realtors (NVM) created Funda as an online real estate platform in 2001 [3]. Since then, Funda has become one of the most innovative digital housing platforms in the world. Archived versions of the Funda website from 2018, which thus correspond to our data, can be found on The Wayback Machine, which was founded by the Internet Archive. See, for instance, the archived version of January 2, 2018 or the archived English language counterpart of October 2, 2018.

The datasets consist of the information (and search) flows of the users of the Funda housing platform website in the first six months of 2018. The geolocation of the platform user determines the origin of the flow while the location of the property that is viewed determines the destination of the flow. The in total 1.1 billion hits (i.e., the full sample dataset), made by users in the Netherlands on listed objects in the Netherlands, aggregate into 148,216 information flows between municipalities. Approximately 95 percent of the hits relate to properties for sale, the remaining 5 percent concern hits on rental objects. The flows are between 382 origin municipalities and 388 destination municipalities; no origin data is observed for six small municipalities (see below). The descriptives for the full sample dataset are found in column 1 of Table 1. The descriptives show that the 1.1 billion hits of the full sample aggregate into 147,903 non-zero flows and 313 zero flows.

**Table 1:** Flow descriptives of the full sample and split by type of platform user.

	Full sample	Account	Mortgage	Telephone	Email service	Message	Viewing	Buyer
Hits ($\times 10^{6}$)	1096.6	244.3	213.8	230.4	118.3	88.2	92.2	4.4
Events ($\times 10^{6}$)	632.8	150.0	125.7	143.1	70.4	54.3	56.7	2.8
Time on site ($\times 10^{6}$ h)	12.6	2.8	2.6	2.9	1.3	1.0	1.1	0.1
Non-zero flows	147,903	143,997	142,317	142,072	135,050	126,757	122,913	43,704
Total flows	148,216	148,216	148,216	148,216	148,216	148,216	148,216	148,216

Hits (percent)	100	22.3	19.5	21.0	10.8	8.0	8.4	0.4
Events (percent)	100	23.7	19.9	22.6	11.1	8.6	9.0	0.4
Time on site (percent)	100	22.1	20.2	22.8	10.6	8.0	8.6	0.4
Non-zero flows (percent)	99.8	97.2	96.0	95.9	91.1	85.5	82.9	29.5

*Notes*: Account, users that have registered and created a user account; Mortgage, users that have done the online mortgage calculation; Telephone, users that have clicked the button for the real estate agent’s telephone number; Email service, users that have signed up for an email service of new listings within their preferences; Message, users that have contacted the real estate agent through an online form; Viewing, users that scheduled a viewing with the online tool; Buyer, users that registered themselves as the buyer of a property. See also: Steegmans and de Bruin [3, p. 11].

### 1.2 Information flows split by user type

Information flow datasets are also available for different types of users. The users are classified based on their website behavior (i.e., their actions). We provide these data as the intentions for using the platform range from serious house searching to fully recreational search. Flow datasets are provided for users that have registered and created a user account, users that have done the online mortgage calculation at least once, users that have clicked the button for the real estate agent’s telephone number, users that have signed up for an email service of new listings within their preferences, users that have contacted the real estate agent through an online form, users that scheduled a property viewing with the online tool, and users that registered themselves as the buyer of a property (although which property that is is not registered) [3]. Columns 2 to 8 of Table 1 show that the number of zero flows increases as the number of hits in the subsamples becomes smaller. Table 1 also shows that the relative size of the subsamples, expressed as a percentage of the full sample, are similar for different measures. Details on these measures, i.e., hits, events and time spent online, will be provided in the methodology section.

### 1.3 Information flows split by device and network domain

Separate flow datasets are also provided for devices and network domain. The devices are split into desktop (including laptops), tablet, and mobile [3]. Table 2 shows that, for the first six months of 2018, the users of the website most often use a computer (almost 50 percent), followed by mobile phones (about 30 percent), and tablets (over 20 percent). Columns 2, 3 and 4 show that the device splits have few additional zero flows. The network domain is split into set (i.e., not not set) and not set. For 15 percent of the cases the network domain is not set, for the remaining 85 percent the network domain falls within one of the other categories identified by Google Analytics (generally, a specific Internet Service Provider). For the ‘not set’ observations the number of non-zero flows increases to 20,453 compared to 127,763 non-zero flows.

**Table 2:** Flow descriptives of the full sample and split by device and network domain.

	Full sample	Desktop	Tablet	Mobile	Set	Not set
Hits ($\times 10^{6}$)	1096.6	504.1	226.3	366.1	932.0	164.6
Events ($\times 10^{6}$)	632.8	319.9	135.0	178.0	537.7	95.1
Time on site ($\times 10^{6}$ h)	12.6	5.5	2.9	4.2	10.8	1.9
Non-zero flows	147,903	147,147	144,971	146,324	147,872	127,763
Total flows	148,216	148,216	148,216	148,216	148,216	148,216

Hits (percent)	100.0	46.0	20.6	33.4	85.0	15.0
Events (percent)	100.0	50.5	21.3	28.1	85.0	15.0
Time on site (percent)	100.0	43.5	23.3	33.2	85.3	14.7
Non-zero flows (percent)	99.8	99.3	97.8	98.7	99.8	86.2

*Notes*: The device categories are: desktop (both desktop computers and laptops), tablet, and mobile. The network domain categories are: set (i.e., not not set) and not set.

### 1.4 Data format and variables

The datasets are formatted as Comma-Separated Values (CSV; encoding ISO 8859-1/Latin-1). Besides, the data are also available as Stata data files (DTA files; encoding version 11/12). Stata statistical software is, for instance, commonly used in economics [5]. The latter also contain variable labels (see Table 4). The full sample information flow data and the flow data for each subsample are stored in separate datasets.

**Table 3:** Datasets full sample and subsamples.

Name dataset	Describtion
Flows_2018H1	Full sample
Flows_2018H1_isNotAnonymous_true	Subsample of users with an account
Flows_2018H1_isNotAnonymous_false	Subsample of users without an account
Flows_2018H1_mortgage_true	Subsample of users who did a mortgage calculation
Flows_2018H1_mortgage_false	Subsample of users who did not do a mortgage calculation
Flows_2018H1_telephone_true	Subsample of users who viewed a real estate agent’s telephone number
Flows_2018H1_telephone_false	Subsample of users who did not view a real estate agent’s telephone number
Flows_2018H1_email_true	Subsample of users who set email notifications
Flows_2018H1_email_false	Subsample of users who did not set email notifications
Flows_2018H1_message_true	Subsample of users who digitally contacted the real estate agent
Flows_2018H1_message_false	Subsample of users who did not digitally contact the real estate agent
Flows_2018H1_viewing_true	Subsample of users who scheduled a listing with the online tool
Flows_2018H1_viewing_false	Subsample of users who did not schedule a listing with the online tool
Flows_2018H1_buyer_true	Subsample of users who registered themselves as the buyer of a property
Flows_2018H1_buyer_false	Subsample of users who did not register themselves as the buyer of a property
Flows_2018H1_device_desktop	Subsample of hits made from computers (desktops and laptops)
Flows_2018H1_device_tablet	Subsample of hits made from tablets
Flows_2018H1_device_mobile	Subsample of hits made from mobile phones
Flows_2018H1_networkdomain_set	Subsample of hits where the network domain was set (i.e., not not set)
Flows_2018H1_networkdomain_not_set	Subsample of hits where the network domain was not set

**Table 4:** Variable description.

Variable name	Type	Label
user_mun_code	String	Municipality code geolocation user (origin)
user_mun_name	String	Municipality name geolocation user (origin)
object_mun_code	String	Municipality code location object (destination)
object_mun_name	String	Municipality name location object (destination)
distance_centr	Continuous	Euclidean distance between municipality centroids
distance_centr_internal	Continuous	Euclidean distance between centroids and approx. for internal flows
mass_users_geo	Integer	Number of unique users origin
mass_objects_obj	Integer	Number of objects destination
flow_hits	Integer	Hits in outflow
flow_events	Integer	Events in outflow
flow_timeonsite	Integer	Time on site in outflow (in seconds)
within_mun	Binary	Within same municipality (1 = yes)
neighbor_mun	Binary	Bordering municipalities (1 = yes)
within_prov	Binary	Within same province but different municipality (1 = yes)
zeroflow	Binary	Zero flow (1 = yes)

The list of datasets is provided in Table 3. The seven user types are each split into two datasets based on whether of not the specific condition is met. For instance, there are users with an account (condition is ‘true’) and users without an account (condition is ‘false’). The same holds for the remaining user types. For these user types the condition should be met at least once to be true. For instance, in the dataset ‘Flows_2018H1_viewing_true.csv’ all users are included that online scheduled a viewing, regardless of the number of viewings that they planned. ‘Flows_2018H1_viewing_false.csv’ includes the users that never used the tool to schedule a property viewing. The device used splits the full sample into three subsamples: desktop (including laptops), tablet, and mobile. The network domain divides the full sample into two categories: set and not set. The set category consists mostly of Dutch Internet Service Providers.

The full sample and subsample datasets have the same structure and contain the same variables. They each contain the origin municipality, the destination municipality, and the hit flow between them. The municipality codes correspond with those of Statistics Netherlands [6], [7] in order to facilitate interoperability. Apart from the hit flow, flows in terms events and time spent are provided. The datasets include the (Euclidean) distance between the centroids of the municipalities, whether flows are internal (within the same municipality), between neighboring municipalities, or between municipalities within the same province. Finally, the datasets include (endogenous) indicators of the size of the origin and destination municipalities: that is, the number of unique users in the origin and the number of objects (i.e., properties) in the destination. The list of variable names, variable types and variable labels can be found in Table 4.

## 2 Experimental Design, Materials and Methods

### 2.1 Funda’s Google Analytics data

Funda uses Google Analytics, Google’s web analytics tool, to registers website users’ activity. The Google Analytics data is thus, as noted before, proprietary data of Funda. Funda monitors the analytics for purposes related to, for instance, platform stability, security, and product development [8]. Google Analytics registers the data at different observation levels: the hit level, the session level, and the user level. A hit is a website interaction where data is sent to Google Analytics. Hits are the lowest observation level. They are divided into events and pageviews. Events can include link clicks, form submissions and video plays while a pageview occurs when a new page is loaded [3], [9]. Hits by the same user are grouped together in a session. After thirty minutes of user inactivity a session is closed. Website activity that follows afterwards is registered in a new session. Session information includes, for example, the user’s device (desktop, tablet or mobile) and the IP address-based geolocation of the user. Sessions, in turn, can be linked to individual users. The users are identified through cookies [8].

The Google Analytics data consist of dimensions and metrics. Dimensions are attributes of the data, metrics are quantifiable measurements. The default dimensions are the same for all Google Analytics customers, among which Funda. The above-mentioned geolocations and the device used are examples of default dimensions. Apart from that, custom dimensions can be added by the Google Analytics customer. For Funda the custom dimensions include information on the property that is viewed, such as the property’s location and whether it is a rental or sale object. Metrics include, for instance, the number of hits within a session and the time spent on the platform.

### 2.2 The origin and destination of hits

The Google Analytics data described above are the basis of the information flow data described in this article. The basis of the flow data is the individual hit. We are interested in the origin of the hit, i.e., the (approximate) user location, and the destination of the hit, i.e., the location of the property that is viewed. The user’s municipality is derived from the IP address-based geolocation, which is registered at the session level in the Google Analytics data.

Google Analytics provides 1181 distinct geolocations (villages, towns, and cities) for our 1.1 billion hits. Google Analytics refers to this geolocation as ‘city’ although it includes, for instance, Biervliet, Retranchement, and Sint Kruis. These villages had 200, 313, and 329 inhabitants on January 1, 2018, respectively [3], [10]. The 1181 distinct Google Analytics geolocations aggregate into 382 (origin) municipalities.^2^ There are no Google Analytics geolocations within the borders of the six remaining municipalities: Ameland, De Marne, Haarlemmerliede en Spaarnwoude, Rozendaal, Schiermonnikoog, and Vlieland. The flows from these municipalities are thus missing; however, it does not mean that these flows are equal to zero. Hits from these municipalities are, likely, incorrectly contributed to other locations instead.^3^

The most important reason to treat the Google Analytics geolocations (i.e., the user locations) somewhat cautiously is because Funda makes use of Google Analytics’ IP anonymization tool. Funda uses this tool in order to comply with the General Data Protection Regulation (GDPR), which protects the data and privacy of individuals in Europe. The anonymization tool replaces the last bits of an IP address with zeros before it is sent to the Google Analytics server [11]. For instance, using dot-decimal notation, the IP address 131.211.209.183 would become 131.211.209.0 before the geolocation is added. For a thorough report on the impact of the Google Analytics IP anonymization tool, see Clifton and Wan [12].

Using the data of Clifton and Wan [12], Steegmans and de Bruin [3] estimate that at the ‘city’ level 66.40 percent of the cases are unaffected by the anonymization tool for users in the Netherlands over time period covered by the flow data. At the municipality level that increases to an accuracy rate of 69.6 percent. As IP addresses are distributed in blocks to Internet Service Providers, accuracy rates show differences amongst them. There remains, for instance, a 98.4 percent municipal accuracy rate after ‘anonymization’ for the Dutch observations where the network domain is ‘not set’ [3].

### 2.3 The role of users

Users play an important role in the construction of the flow data. First, their geolocation determines the origin of the flow. Second, user actions are used to generate the user type (subsample-based) datasets. For the latter, hits and sessions from repeat visits have to be assigned to the correct user. This requires a unique user identifier. The Google Analytics user identifier (fullVisitorId) and the Funda user identifier for logged-in users allow for this. Importantly, anonymity is guaranteed as the fullVisitorId cannot be matched to other sources; it has only purpose within Funda’s Google Analytics environment. Furthermore, no personally identifiable information (PII) is included; full nor anonomized IP addresses are stored in Google Analytics. Only the corresponding geolocation is registered. Apart from that, we do not have access to user accounts, which are likely to contain PII for at least part of the users. Similarly, houses and apartments have a unique Funda ID so that address information of the objects viewed does not have to be linked. For the registered buyers we only observe that an (unidentified) property is being registered. Users are free in their choice to register themselves as buyer or not.

### 2.4 Variable construction and measurement

Hits are the basis of the information flows described in this article. Nevertheless, two flow alternatives are available. The first are the flows in terms of events, a subcategory of the hits. Both hits and events have been described at the beginning of this section. The second alternative are the flows in terms or time spent viewing properties. In other words, the total time that users from a given municipality spent viewing properties from another municipality (or their own). This variable, however, contains a particularly large measurement error. The default Google Analytics ‘time on site’ variable provides information at the session level. It is impossible to derive the time flow data from this measure. After all, the time spent must be contributable to individual properties, instead of sessions in which a multitude of properties are viewed, for meaningful flows to be constructed. Therefore, we must rely on the time stamps of individual hits that relate to a given property. In this approach, however, it is not possible to unequivocally determine when the view of a property ends. Furthermore, users switching between properties/webpages also distort the measurements.

The municipalities are classified by their Statistics Netherlands (CBS) identifier code, the ‘GM code’ [6]. The GM code is used because it facilitates matching the flow datasets to public data from Statistics Netherlands. Most notably, shape files – for the creation of maps – and municipality figures, such as population statistics and surface areas [7].

The distance variable is measured as the Euclidean distance between the centroids of a municipality pair. The centroids are RD (*Rijksdriehoek*) coordinates from the spatial reference system, industry standard EPSG:28992 [13], that is used by the Cadastre and government organisations in the Netherlands. As the *x* and *y* coordinates are expressed in meters the distances follow directly from them. For internal flows, i.e., flows within a municipality, the distance between the centroids would be zero. As this underestimates the true (internal) distance, we use the Head and Mayer approximation [14] instead. The internal distance is therefore given by: $\frac{2}{3}\sqrt{A/\pi }$ where A is the surface area of the municipality. The internal distance approximations are thus used when the origin and destination municipality are the same.

## Ethics and Privacy Statement

Funda has to comply with the General Data Protection Regulation (GDPR) when collecting website analytics. According to Funda, Google Analytics is set up “as privacy friendly as possible” [15]. The privacy friendly settings include, for instance, the use of Google Analytics’ IP anonimization tool, which use is strongly recommended by the Dutch Data Protection Authority [16]. The importance of data protection and privacy is illustrated by the fact that the Dutch Data Protection Authority considers the ‘anonimized’ IP address, thus excluding the last 8 bits for an IPv4 address, personal information nonetheless [16]. Although the (anonimized) IP address is used to determine the user geolocation, the IP address itself is not registered in the (underlying) data that we use to generate the flow datasets. The information flow data described here, although based on user-generated data, contains no personally identifiable information (PII) whatsoever. The information flow data have been aggregated into municipal flows, which cannot be traced back, in any way, to individual users. Finally, Funda authorized the publication of the flow data that accompany our related research article and which are described in detail in this data article.

## CRediT authorship contribution statement

**Joep Steegmans:** Conceptualization, Data curation, Validation, Writing – original draft, Writing – review & editing. **Jonathan de Bruin:** Data curation, Software, Validation.

## Declaration of Competing Interest

The authors declare that they have no known competing financial interests or personal relationships which have, or could be perceived to have, influenced the work reported in this article.

## Appendix A Supplementary materials

Supplementary Data S1 Supplementary Raw Research Data. This is open data under the CC BY license http://creativecommons.org/licenses/by/4.0/ Supplementary Data S1

## Footnotes

1 As the basics of the data and the dataset construction method are also covered in the data section of the related research article [3], the content of the description may occasionally overlap.

2 We rely on the municipality borders as specified in 2017 for consistency reasons. Information on these municipality borders is available from Statistics Netherlands [6], [7].

3 See also: Steegmans and de Bruin [3].

## References

[1] K. Sato, An inside look at Google BigQuery. White Paper WP2031-1210, 2012. Available at: https://cloud.google.com/files/BigQueryTechnicalWP.pdf. Accessed January 21, 2021.

[2] J. Steegmans, J. de Bruin, Online housing search dataset: information flows of real estate platform users [dataset]. Mendeley Data, V1, doi:10.17632/rtn8t47t6j.1, 2021. PMC8411214 34504913

[3] Steegmans J., de Bruin J. (2021). Online housing search: a gravity model approach. PLoS One.

[4] Head K., Mayer T. (2014). Handbook of International Economics.

[5] StataCorp LLC. Disciplines: Economics, 1996–2021. https://www.stata.com/disciplines/economics/. Accessed August 9, 2021.

[6] Statistics Netherlands. Toelichting wijk- en buurtkaart 2015, 2016 en 2017 [Explanation district and neighborhood map 2015, 2016 and 2017]. CBS Paper, August, 2017a. https://www.cbs.nl/-/media/_pdf/2017/36/2017ep37-toelichting-wijk-en-buurtkaart-2017.pdf. Accessed December 12, 2020.

[7] Statistics Netherlands. Wijk- en buurtkaart 2017 [District and neighborhood map 2017; Data on digital geometry of municipality borders]. Statistics Netherlands, Geographic Data, 2017b. https://www.cbs.nl/nl-nl/dossier/nederland-regionaal/geografische-data/wijk-en-buurtkaart-2017. Accessed December 12, 2020.

[8] Funda. Cookies: Cookievoorkeuren [Cookies: Cookie preferences]. Last updated: September 10, 2018. https://www.funda.nl/en/cookiebeleid/. Accessed April 2, 2019.

[9] Google Analytics, Glossary. Last updated: unknown, s.a. https://support.google.com/analytics/topic/6083659. Accessed April 2, 2019.

[10] Municipality of Sluis. Sluis in cijfers [Municipality of Sluis in numbers], 2018. Last updated: unknown. https://www.gemeentesluis.nl/Bestuur_en_Organisatie/Gemeente_in_beeld/Sluis_in_cijfers. Accessed August 1, 2019.

[11] Google Analytics. IP Anonymization (or IP masking) in Analytics. Analytics Help > Data privacy and security, 2020. https://support.google.com/analytics/answer/2763052. Accessed December 9, 2020.

[12] B. Clifton, H. Wan, Anonymize IP GA Impact Assessment 2.7. Technical report, June, 2018. https://brianclifton.com/research/Anonymize_IP_GA_Impact_Assessment.html. Accessed January 30, 2019.

[13] EPSG. EPSG:28992. Amersfoort / RD New – Netherlands - Holland - Dutch. Coordinate Systems Worldwide, Revision date: 2005-05-27, 2005. Available at: https://epsg.io/28992. Accessed January 21, 2021.

[14] Head K., Mayer T. (2010). The Gravity Model in International Trade: Advances and Applications. Editors: Van Bergeijk and Brakman.

[15] Funda. Cookiebeleid [Cookie policy]. Last updated: March 26, 2020. https://www.funda.nl/cookiebeleid/. Accessed December 12, 2020.

[16] Dutch Data Protection Authority. Handleiding privacyvriendelijk instellen van Google Analytics [Manual privacy friendly settings of Google Analytics]. Autoriteit Persoonsgegevens [Dutch Data Protection Authority], August 15, 2018. https://autoriteitpersoonsgegevens.nl/sites/default/files/atoms/files/138._handleiding_privacyvriendelijk_instellen_google_analytics_aug_2018.pdf. Accessed December 10, 2020.

